# Migraine without aura is not associated with incomplete circle of Willis: a case–control study using high-resolution magnetic resonance angiography

**DOI:** 10.1186/1129-2377-15-27

**Published:** 2014-05-10

**Authors:** Shabnam Ezzatian-Ahar, Faisal Mohammad Amin, Hayder Ghani Obaid, Nanna Arngrim, Anders Hougaard, Henrik B W Larsson, Messoud Ashina

**Affiliations:** 1 Danish Headache Center, Department of Neurology, Glostrup Hospital, University of Copenhagen, Nordre Ringvej 57, Glostrup DK-2600, Denmark; 2 Diagnostic Department, Glostrup Hospital, University of Copenhagen, Nordre Ringvej 57, Glostrup, DK-2600, Denmark

**Keywords:** Circle of Willis, Migraine without aura, Cerebral arteries, Stroke

## Abstract

**Background:**

The circle of Willis is an important source of collateral blood flow to maintain adequate cerebral perfusion, particularly in the posterior circulation. Some studies report a relationship between incomplete circle of Willis and migraine, whereas other studies show no difference between the prevalence of incomplete circle of Willis in migraineurs and controls. In the present study we compared the prevalence of incomplete circle of Willis in female migraine patients without aura to female healthy non-migraine controls.

Using 3-Tesla magnetic resonance angiography we recorded three-dimensional time-of-flight angiograms in 85 female participants (48 migraine patients without aura [median age 28 years] and 37 healthy controls [median age 25 years]). The images were subsequently analysed blindly by a neuroradiologist to detect incomplete circle of Willis.

**Findings:**

We found no difference between the prevalence of incomplete circle of Willis in patients, 20/47 (43%), and controls, 15/37 (41%), *p* = 0.252. Post hoc analysis showed a significant relationship between age and prevalence of incomplete circle of Willis, *p* = 0.003.

**Conclusion:**

We found no relationship between migraine without aura and incomplete circle of Willis.

## Introduction

Migraine patients are at increased risk of ischemic vascular disease [1]. The risk of ischemic stroke is roughly doubled in patients with migraine [2]. Furthermore, some studies reported a positive correlation between the risk of ischaemic stroke and frequency of migraine attacks. The mechanisms that link migraine to ischemic vascular disease are unknown [3]. It seems that cerebral infarcts preferentially affect posterior circulation [4,5]. The presence of the circle of Willis anomalies may contribute to decrease of cerebral blood flow in response to cortical spreading depression [6]. The circle of Willis is an important source of collateral blood flow to maintain adequate cerebral perfusion, in particular the posterior. Human studies show large anatomical variability in the general population [7]. Most recently, Cucchiara and colleagues [8] found significant higher prevalence of an incomplete circle of Willis in migraine with aura (71%) and a strong trend (*p* = 0.08) in patients without aura (67%) compared to controls (51%). Based on these data the authors suggested that migraine with aura might differ from migraine without aura in terms of vascular physiology [8]. This study included patients elder than 25 years. Age is negatively correlated with arterial diameter in the circle of Willis [7], which causes that the smallest arteries may not be visualized unless the image resolution is accordingly increased. In addition, this study used a clinic-based control group, which potentially could have biased the outcome. These factors could potentially influence the outcome on differences between migraine without aura patients and controls. Therefore, in the present study we studied a large number of female migraine patients without aura and compared them to healthy volunteers without known risk factors for cerebrovascular diseases using high-resolution time-of-flight magnetic resonance angiography at 3-Telsa. We hypothesized that circle of Willis is more frequent in patients compared with the healthy volunteers.

## Material and methods

### Participants and study design

**Figure 1:**
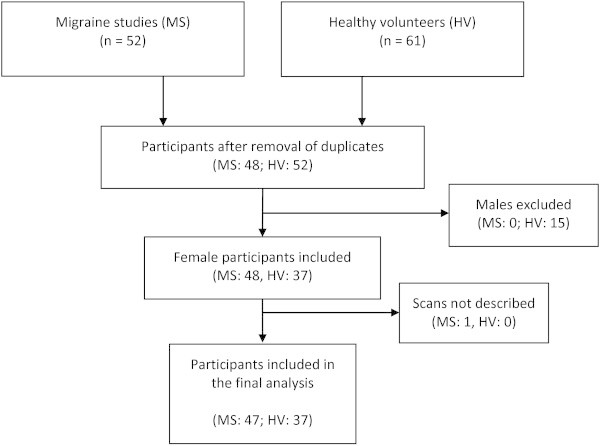
Flowchart of the study.

For this study, we pooled all magnetic resonance angiography images of female participants recorded at baseline in several previous studies of migraine without aura patients and healthy volunteers at the Danish Headache Center (Glostrup, Denmark) between 2007 and 2013 (Figure 1). None of the participants had a headache or used painkillers or triptans at least 48 h prior to magnetic resonance angiography scans. Moreover, none of the participants suffered from a serious somatic disease, particularly cardiovascular or cerebrovascular disease; were pregnant or nursing; had any contradiction for magnetic resonance imaging scan (i.e. metal in the body or claustrophobia). Smoking was an exclusion criterion in all studies. Exclusion criteria for the migraine patients were chosen to avoid factors, which could, potentially, affect the imaging outcome. For instance, vascular comorbidity and altered cerebral blood flow are factors that affect the MR signal. Additionally, none of the healthy volunteers had any headache more than once per month. Migraine patients fulfilled the IHS criteria [9] for migraine without aura and they were recruited from the outpatient clinic at the Danish Headache Center or via announcements on a Danish website for recruitment of volunteers for biomedical research (http://www.forsoegsperson.dk). Five out of 48 patients were recruited from the outpatient clinic. All healthy volunteers were recruited from http://www.forsoegsperson.dk. The regional ethical committee of Copenhagen, Denmark, approved all included studies and a written informed consent was obtained from participants. The studies were conducted in accordance with the Helsinki Declarations.

### MRA acquisition

In all studies a 3.0 Tesla Philips Achieva machine (Philips Medical Systems, Best, Netherlands) was used to acquire single-slab three-dimensional time-of-flight magnetic resonance angiography of the circle of Willis with the following parameters: field of view, 200 × 200 × 74 mm^3^; matrix size, 800 × 406; acquired voxel resolution, 0.25 × 0.49 × 1.00 mm^3^; reconstructed resolution, 0.20 × 0.20 × 0.50 mm^3^; repetition time, 25 ms; echo time 3.5; flip angle 20 degrees; sense factor 2; four chunks; acquisition time, 9 min 3 seconds.

### Image analysis

**Figure 2:**
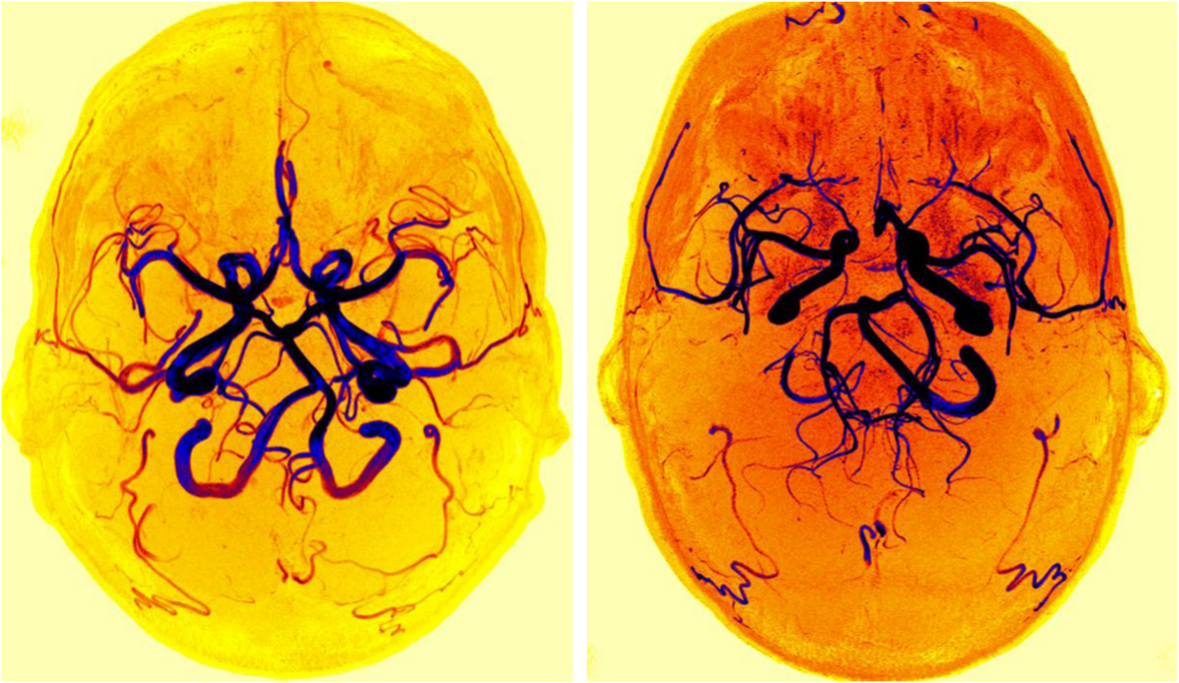
Axial maximum intensity projection images of a complete (left side) and an incomplete (right side) circle of Willis from one patient.

Magnetic resonance angiography data were imported in DICOM format to the free DICOM viewer K-PACS for Windows (version 1.6.0) by a senior radiologist (HGO) in blinded and randomized fashion. The radiologist assessed the circle of Willis morphology once and reported if the circle was complete or incomplete. Assessment of the circle of Willis was done according to Krappe-Hartkamp et al. [7] and Cucchiara et al. [8]. Maximum intensity projections (Figure 2) and source images (with three-dimensional reconstruction) were both used for circle of Willis classification. Circle of Willis was thus classified as 1) complete (all component vessels of both anterior and posterior parts of the circle of Willis visible and ≥ 0.8 mm diameter) or 2) incomplete (either anterior or posterior parts of the circle of Willis incomplete). It is difficult to assess the smaller communicating arteries on two-dimensional images and it may require repeated assessments or different observers to be confident. However, it is much easier to detect the presence or absence of these small arteries using the three-dimensional reconstruction mode. We, therefore, decided that only one radiologist would assess the images.

### Statistics

The primary endpoint of the study was to compare the prevalence of an incomplete circle of Willis between migraine patients without aura and healthy controls. A multiple logistic regression analysis was performed to investigate whether there was a simple relationship between incomplete circle of Willis and migraine without aura or if the participant ages played a role. Circle of Willis was selected as the dependent variable and the diagnosis and age as explanatory variables. Statistical analyses were performed using IBM SPSS Statistics for Mac (version 20.0.0), and the level of significance was accepted at 5%.

## Findings

We identified 85 female participants, of whom 48 suffered from migraine without aura (median age, 28 years [range, 20–58 years]) and 37 were healthy controls (median age, 25 years [range, 20–34 years]). The magnetic resonance angiography image for one patient could not be assessed because of motion blurs.

### Circle of Willis and migraine without aura

**Figure 3:**
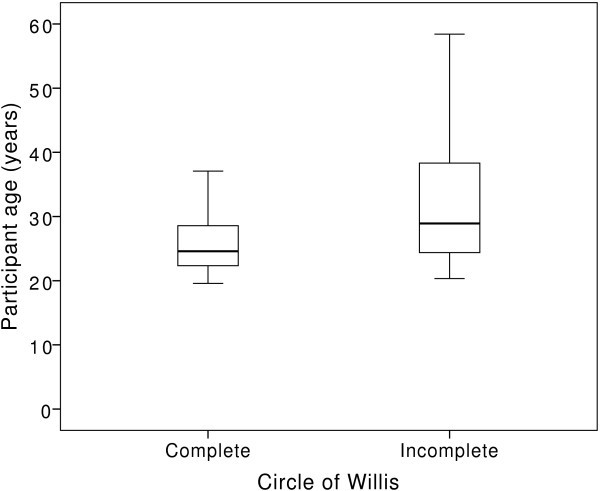
**The prevalence of an incomplete circle of Willis showed a significant relationship with participant age (*p*** **= 0.003).**

We found no difference between the prevalence of incomplete circle of Willis in patients, 20/47 (43%), and controls, 15/37 (41%), *p* = 0.252. In addition, we found a significant relationship between age and prevalence of incomplete circle of Willis, *p* = 0.003 (Figure 3).

## Discussion

The main finding of the present study is that migraine without aura is not associated with a higher prevalence of an incomplete circle of Willis compared to healthy controls (43% versus 41%). Human studies show large anatomical variability in the general population. Using magnetic resonance angiography of healthy volunteers, the reference value for morphologic variants was reported as high as 58% [7]. Our study confirms a recently published study by Cucchiara and colleagues [8] who also found no difference in the prevalence of incomplete circle of Willis between migraine without aura patients and controls. A retrospective study showed no difference in circle of Willis prevalence between migraine patients and healthy volunteers [10], whereas two other studies reported circle of Willis anomalies to be significant more frequent in patients with and without aura [11,12]. The most recently published study by Cucchiara and colleagues [8] found no difference in circle of Willis anomalies between migraine without aura and healthy subjects (67% versus 51%, *p* = 0.08). The participants were recruited from a neurological clinic and by advertisement. Participants referred to a neurological clinic may not be labelled as being completely healthy. This can partly explain the slightly higher prevalence of incomplete circle of Willis reported by Cucchiara and colleagues [8] compared to the present study. In our study, the patients were young and were excluded if they suffered from other serious medical disorders. Increasing age is positively correlated with an incomplete circle of Willis. The patients in our study did not suffered from less severe migraine, but had less co-morbidity, which may affect the outcome. The strength of our study is also that we included only female patients and controls. Furthermore, the controls were completely healthy volunteers without other diseases, headaches or use of any kind of daily medication (except oral contraceptives). The proposed relationship between migraine and an incomplete circle of Willis is that it might cause decrease in regional cerebral blood flow. If the brain is hyperexcitable the incomplete circle of Willis may not provide satisfactory blood supply, which may result in ischemia and cortical spreading depression [6]. This theory could be plausible in patients with aura, but in patients without aura it is even more speculative suggesting “silent” cortical spreading depression [13]. In conclusion, in agreement with previous studies, we found no relationship between female migraineurs without aura and an incomplete circle of Willis.

## Competing interests

The authors declare that they have no competing interests in relation to this study.

## Authors’ contributions

All authors contributed to protocol development, study design, drafting and revision of the paper. FMA, NA and AH further contributed with data acquisition, data processing and statistics. HGO further contributed with data analysis. SEA, FMA and MA further contributed with data interpretation. All authors read and approved the final manuscript.
